# A multi-trait genome-wide association study of coronary artery disease and subclinical atherosclerosis traits

**DOI:** 10.21203/rs.3.rs-6456056/v1

**Published:** 2025-04-21

**Authors:** Paul de Vries, Natalie Hasbani, Adam Heath, Chani Hodonsky, Julie Hahn, Devendra Meena, Haojie Lu, Abbas Abbas Dehghan, Maryam Kavousi, Benjamin Voight, Patricia Peyser, Alanna Morrison, Themistocles Assimes, Scott Damrauer, Clint Miller

**Affiliations:** Human Genetics Center; The University of Texas Health Science Center at Houston; The University of Texas Health Science Center at Houston; University of Virginia; The University of Texas Health Science Center at Houston; Imperial College London; Erasmus University Medical Center; Imperial College London; Erasmus MC; University of Pennsylvania; Department of Epidemiology, School of Public Health, University of Michigan; The University of Texas Health Science Center at Houston; Stanford University School of Medicine; University of Pennsylvania; University of Virginia

## Abstract

Measures of subclinical atherosclerosis, such as coronary artery calcification (CAC) and carotid intima-media thickness (CIMT), reflect the underlying pathophysiology of coronary artery disease (CAD) and are genetically correlated with CAD and related risk factors. Leveraging summary statistics from genome-wide association studies of CAD, CIMT, CAC, type 2 diabetes, low-density lipoprotein cholesterol, and systolic blood pressure, we performed 15 separate multi-trait GWAS to identify shared susceptibility loci and elucidate the pleiotropic architecture underlying atherosclerosis. We identified 442 shared risk loci across all analyses that met an experiment-wide Bonferroni threshold of 3.3 × 10^−9^, uncovering 195 novel atherosclerosis loci. Multi-trait colocalization confirmed a shared causal signal in 25 shared novel loci for atherosclerosis. Trait-eQTL colocalization identified evidence of a shared causal signal in arterial, subcutaneous adipose, and cardiac tissues, implicating genes such as *PRRX2*, *BNC2*, *CLIC4*, *SCAI*, and *PPP6C*, and pathways related to vascular remodeling, inflammation, and metabolic regulation.

## Introduction

Coronary artery disease (CAD) is a complex disease process involving a convergence of environmental and genetic risk factors. Several underlying heritable traits exist which indicate the presence of atherosclerosis prior to the clinical manifestation of CAD, including coronary artery calcification (CAC) and carotid intima- media thickness (CIMT). Recent genetic studies also provide abundant evidence of pleiotropy among risk factors with approximately 50% of identified CAD risk loci associating with underlying clinical risk factors.^1–11^

Genome-wide association studies (GWAS) for direct and indirect measures of atherosclerosis as well as associated risk factors such as systolic blood pressure (SBP), low-density lipoprotein cholesterol (LDL-C), and type 2 diabetes (T2D) have identified hundreds of susceptibility loci.^10–20^ However, the discovered loci collectively only explain a fraction of the heritability of these phenotypes, suggesting that additional associated susceptibility loci remain to be discovered.^21^ Combining information from the shared genetic architecture between atherosclerosis measures and clinical risk factors may help identify new susceptibility loci with shared underlying biology. Statistical approaches that facilitate the joint analysis of multiple correlated traits have been developed that increase the power to detect loci that are pleiotropically associated with more than one trait.^22,23^ These methods have thus far uncovered many shared susceptibility loci for cardiometabolic phenotypes and provided unique insight into shared genetic pathways.^24–26^

No studies to date have used multi-trait approaches to evaluate both subclinical and clinical atherosclerosis phenotypes along with risk factors. Joint analysis of clinical CAD with subclinical atherosclerosis traits and related risk factors may uncover key susceptibility loci and pleiotropic mechanisms contributing to the development of CAD. Here, we report a multi-stage, multi-trait GWAS of atherosclerosis-related phenotypes and selected cardiovascular risk factors designed to provide deeper insights into the shared genetic architecture underlying atherosclerosis and enhance power for locus discovery.

## Results

### Cross-trait analysis for atherosclerosis and related risk factors

**Figure 1:**
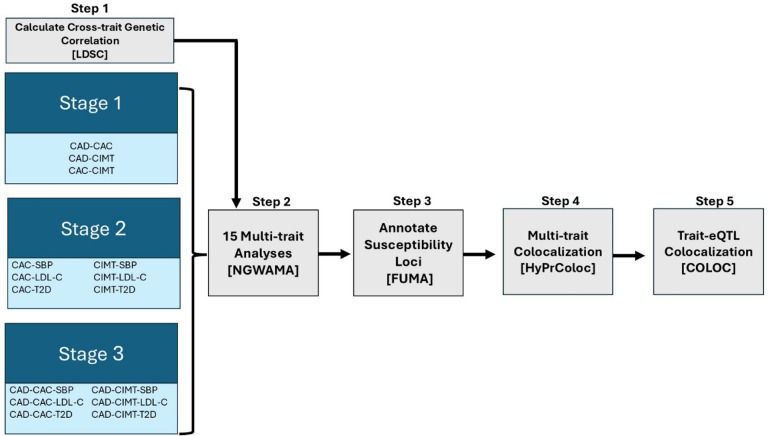
Overview of multi-stage, multi-trait genome-wide association study Figure 1 provides an overview of the multi-stage, multi-trait genome-wide association study. Stage 1 trait combinations were coronary artery disease (CAD)-coronary artery calcification (CAC), CAD-carotid intima- media thickness (CIMT), and CAC-CIMT. Stage 2 trait combinations were CAC or CIMT with systolic blood pressure (SBP), low- density lipoprotein cholesterol (LDL-C), or type 2 diabetes (T2D). Stage 3 trait combinations were CAD and CAC or CAD and CIMT with SBP, LDL-C or T2D. We first conducted linkage disequilibrium score regression using LD Score (LDSC) v1, then conducted 15 multi-trait genome-wide association analyses in N-weighted multivariate genome-wide association meta-analysis (N-GWAMA), annotated using Functional Mapping and Annotation of GWAS (FUMA) to define risk loci, prior to performing trait-trait colocalization in HyPrColoc and trait-eQTL colocalization using COLOC packages in R. Abbreviations: CAC, coronary artery calcification; CAD, coronary artery disease; CIMT,carotid intima-media thickness; FUMA, Functional Mapping and Annotation of GWAS; eQTL, expression quantitative loci; GWAS, genome-wide association study; LDL-C, low- density lipoprotein cholesterol; N-weighted multivariate genome-wide association meta-analysis,N-GWAMA; SBP, systolic blood pressure, SNP, single nucleotide polymorphism

**Figure 2:**
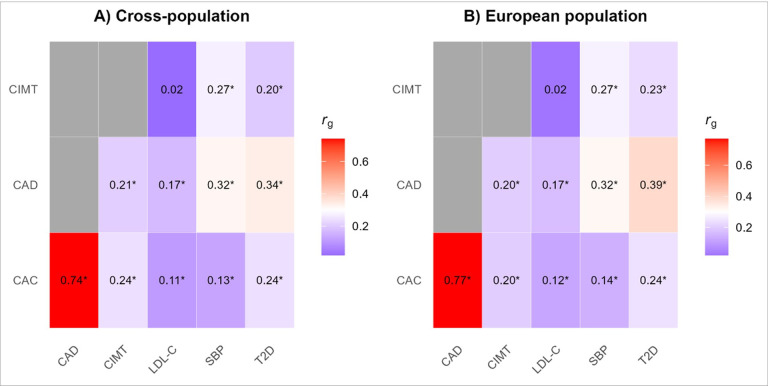
Genetic correlations across atherosclerosis and associated risk factors Figure 2 is a heatmap of genetic correlation coefficients across atherosclerosis and risk factor traits. Dark red refers to a strong positive genetic correlation while dark blue indicates no correlation. Asterisk indicates a significant genetic correlation (P<0.05). Panel A provides genetic correlation coefficients using cross-population summary statistics for coronary artery disease (CAD), carotid itima media thickness (CIMT), coronary artery calcification (CAC), low density lipoprotein cholesterol (LDL-C) and type 2 diabetes (T2D). Panel B provides European-specific genetic correlation coefficients for studies as were provided for CAC, CIMT, LDL-C, systolic blood pressure. Abbreviations: CAC, coronary artery calcification; CAD, coronary artery disease; CIMT, carotid intima media thickness, LDL-C, low density lipoprotein cholesterol; SBP, systolic blood pressure, T2D, type 2 diabetes

We conducted a multi-stage, multi-trait analysis using GWAS summary statistics collected from 3 different measures of atherosclerosis (CAD, CAC, and CIMT) and 3 risk factors (T2D, LDL-C, SBP, Fig. 1, **Supplemental Table 1**).^10,12,14,16,17,19,20^ First, we conducted cross-trait linkage disequilibrium (LD) score regression using LD Score v1.0 (LDSC) to estimate genetic correlation, and shared heritability for each of the 12 pairs of traits.^27^ Multi-ancestry GWAS summary statistics were available for all traits except SBP where summary statistics were only available from those of European ancestry at the time of this analysis. Analyses were performed using the 1000 Genomes European LD reference panel.^28^ All atherosclerosis and related risk factor traits were significantly (P < 0.05) genetically correlated with CAD (Fig. 2). Both CAC and CIMT were significantly genetically correlated with T2D and SBP. CAC was also significantly genetically correlated with LDL-C, but CIMT was not. The strongest observed genetic correlation across all traits was between CAC and CAD [*r*_*g*_=0.74 ± 0.05 (standard error)], followed by CAD and T2D [*r*_*g*_=0.34 ± 0.02]. For studies that had ancestry-specific datasets available, we repeated the analysis using European-only datasets and found the genetic correlations to be similar with only slight variations in the magnitude and precision of the estimates (**Supplemental Table 2**, Fig. 2).

#### Multi-trait GWAS

We conducted a multi-stage, multi-trait GWAS using N-weighted multivariate genome-wide association meta-analysis (N-GWAMA).^23^ N-GWAMA applies the estimates from cross-trait LD-score regression to re-weight test statistics from single-trait GWAS summary statistics, by sample size and estimated heritability, while adjusting for genetic covariance across traits. We conducted 15 multi-trait analyses (Fig. 1) across three stages to systematically assess the genetic architecture of atherosclerosis-related traits and risk factors. Stage 1 focused on atherosclerosis traits (CAD, CAC, CIMT) to capture shared genetic associations within clinically relevant disease endpoints. Stage 2 incorporated subclinical traits and selected risk factors (e.g., LDL-C, SBP, T2D) to assess their independent and joint contributions. Stage 3 integrated both approaches, combining atherosclerosis traits with risk factors (e.g., CAD-CAC-SBP) to dissect the genetic pathways underlying disease progression from subclinical to clinical disease. By structuring our analysis in this manner, we aimed to maximize power for variant discovery while mitigating dilution effects from unassociated phenotypes. This approach also enabled us to differentiate variants associated with distinct biological pathways contributing to atherosclerosis development.

We used Functional Mapping and Annotation of GWAS (FUMA) to annotate the summary results generated from N-GWAMA.^29^ We identified 1,177 multi-trait risk loci at a GWAS significance threshold of 5×10^−8^, of which 948 met the experiment-wide Bonferroni-corrected significance threshold of 3.3×10^−9^ (5×10^−8^÷15) across all stages of analysis (**Supplemental Fig. 1–3**). A risk locus was considered novel for atherosclerosis if the lead variant did not meet the genome-wide significance threshold in any of the initial single-trait summary statistics for CAC, CAD, or CIMT and was located more than 500 kb away from a previously reported genome-wide significant variant in the GWAS Catalog.^30^ For Stage 2 analyses which were restricted to subclinical atherosclerosis traits, a locus was considered novel only if it had not been reported in the initial CAD summary statistics and was also more than 500 kb away from any genome-wide significant variant for CAD. This approach ensured that all reported loci were novel for all measures of atherosclerosis regardless of analysis stage.

We identified 173 significant loci during Stage 1 analyses. Most of the significant loci had known associations with at least one atherosclerosis trait (CAD, CAC, or CIMT) (**Supplemental Table 3**). Only one locus, rs472784 in *DLG2* (P = 2.0×10^−9^), from the CAD-CIMT analysis was novel for atherosclerosis. Similarly, in Stage 2 analyses, 451 experiment-wide significant loci were identified and 210 were novel atherosclerosis loci (**Supplemental Table 3, Supplemental Table 4**). Of these, 10 loci were also novel for the included risk factor. Finally, during Stage 3 analyses, we identified 324 significant loci with 115 novel loci for atherosclerosis. There were 17 significant loci that were also novel for the selected risk factor in the analysis (**Supplemental Table 3, Supplemental Table 5**).

**Figure 3:**
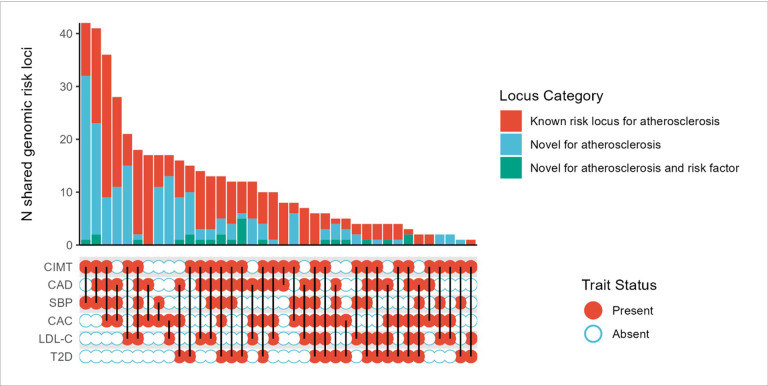
Summary of shared risk loci identified in multi-trait genome-wide association analysis Figure 3 is an upset plot summarizing the shared loci identified in our multi-trait genome- wide association study (GWAS). The y-axis contains the counts for the number of shared combined genetic risk loci across stages of analysis. Each individual risk locus was identified in a multi-trait analysis of atherosclerosis and select risk factor traits. Lead variants from an individual genetic risk loci were collapsed into a single shared risk locus if lead variants were within 500 kb of each other. Lines connect groups of traits within a shared risk locus. A locus was considered novel for atherosclerosis if the lead variant did not meet the GWAS significance threshold (P=5×10^−8^) in the single-trait atherosclerosis summary statistics and was more than was ±500 kb away from a GWAS significant variant in the GWAS Catalog. When the analysis was restricted to subclinical atherosclerosis traits, a locus was considered novel for atherosclerosis if it was more than ±500 kb away from a GWAS significant variant for coronary artery disease. Abbreviations: CAC, coronary artery calcification; CAD, coronary artery disease; CIMT, carotid intima-media thickness; GWAS, genome-wide association study; LDL-C, low- density lipoprotein cholesterol; SBP, systolic blood pressure, T2D, type 2 diabetes

To identify shared and unique loci across all multi-trait analyses, we merged characterized loci into a single shared genomic locus if the lead variants from different analyses were within 500 kb of each other. For each shared locus, the variant with the most significant P-value across analyses was designated as the shared lead variant. Thus, the 1,177 multi-trait risk loci at GWAS significance threshold of 5×10^−8^ collapsed into 535 shared loci across all stages of analysis, with 442 containing at least one experiment-wide significant multi-trait locus (Fig. 3, **Supplemental Table 6**). Most of the 442 significant shared risk loci were known genomic regions that have previously been associated with atherosclerosis, with 195 shared risk loci that were novel for atherosclerosis. There were 25 loci that were also novel for a selected risk factor in the multi-trait analysis. Half of the novel atherosclerosis loci were identified in analyses restricted to subclinical atherosclerosis (101/195 = 51%). Overall, there were 60 novel atherosclerosis loci associated with CIMT, 27 loci associated with CAC, and 14 novel atherosclerosis loci overlapping with CAC and CIMT. The remaining novel atherosclerosis loci were identified in analyses with CAD (N = 94). There were 41 novel atherosclerosis loci shared between CAD and CIMT, 30 shared between CAC and CAD and 23 shared across all atherosclerosis traits (CAD, CAC, and CIMT). For novel atherosclerosis loci, 4 regions overlapped the most frequently across all stages of multi-trait analyses (nearest gene: *BNC2*, *GPATCH2*, *INSR*, *JAZF1*).

Most of the experiment-wide significant loci were identified in multi-trait analyses which included SBP (Fig. 3). Distinct groups of shared genomic regions were identified across atherosclerosis traits. There were 50 shared loci shared across SBP, CIMT, and CAD, 58 shared loci that included just CIMT-SBP, 37 shared across all atherosclerosis traits and SBP and 35 shared loci that included CAC-CAD-SBP. Similar patterns were noted with both remaining risk factor traits with various trait combinations identifying important pleiotropic genomic regions for atherosclerosis. Six shared loci that were shared across all traits in the analysis, all with known associations with atherosclerosis (nearest genes: *MAT2A*, *IRS1*, *STAG1*, *PVRL2*, *OPRL1*, *ARVCF*).

### Trait-trait and Trait- eQTL colocalization

**Figure 4:**
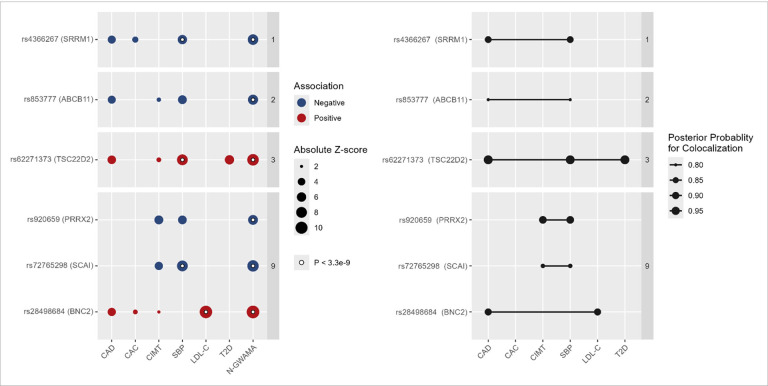
Novel atherosclerosis loci with evidence of trait-trait colocalization Figure 4 summarizes the multi-trait genome-wide association study (GWAS) and multi-trait colocalization performed to identify novel loci associated with atherosclerosis. Panel A provides the results of multi-trait GWAS. The *x*-axis denotes the single trait GWAS and N-weighted genome- -wide association meta-analysis (N-GWAMA) results, and the *y*-axis denotes the pleiotropic independent lead variants at each locus with the nearest gene in parenthesis. The presence of a dot indicates a trait involved in the N-GWAMA. The size of each point denotes the absolute *z*-score for each trait. Associations exceeding the Bonferroni threshold are denoted with a white circle. Variants are grouped by chromosome. Panel B presents the results of multi-trait colocalization. The *x*-axis denotes all atherosclerosis and risk factor traits. The *y*-axis represents the lead variant at each independent locus identified in the multi-trait GWAS. Lines connect groups of traits with evidence of colocalization at a given locus. The size of each point represents the posterior probability for colocalization. Evidence for colocalization was determined based on the default variant-specific regional and alignment priors (P_R_*=P_A_*=0.5), with colocalization identified when P_*R*_P_*A*_≥0.25. Results are restricted to those with P_*R*_P_*A*_A>0.80. Abbreviations: CAC, coronary artery calcification; CAD, coronary artery disease; CIMT, carotid intima-media thickness; GWAS, genome-wide association study; LDL-C, low- density lipoprotein cholesterol; N-GWAMA, N-weighted genome- -wide association meta-analysis; SBP, systolic blood pressure, T2D, type 2 diabetes

Multi-trait colocalization was conducted using HyPrColoc, a Bayesian divisive clustering algorithm designed to identify shared causal signals within a genomic region.^22^. Evidence for colocalization was determined based on the default variant-specific regional and alignment priors (P_R_*=P_A_*=0.5) with colocalization identified when P_*R*_P_*A*_≥0.25. Strong evidence of colocalization across traits was defined as P_R_P_A_ ≥ 0.80. Overall, 164 significant shared risk loci identified in the multi-trait analysis also had evidence of multi-trait colocalization with a measure of atherosclerosis in HyPrColoc. Overall, multi-trait GWAS analyses that included SBP also colocalized the most frequently with the respective atherosclerosis traits (N = 164), followed by LDL-C (N = 93), and T2D (N = 36). There were 25 novel atherosclerosis loci with evidence of colocalization (**Supplemental Table 7**). Of the 25 shared novel atherosclerosis loci with evidence of colocalization, 7 analyses strongly colocalized with P_R_P_A_ ≥0.80 (nearest gene: *BNC2*, *SCAI*, *TSC22D2*, *SRRM1*, *ABCB11*, *PRRX2* [Figure 4]).

Finally, for loci that colocalized in the trait-trait analysis, we performed trait- expression quantitative loci (eQTL) colocalization using eQTL data from GTEX v8 using the COLOC package in R.^31,32 33^ Using summary statistics from the multi-trait GWAS for corresponding trait-trait pairwise colocalization, we focused our analysis on a subset of GTEx v8 tissues selected based on their biological relevance to the studied traits (**Supplemental Table 8**). Evidence for colocalization was defined as a posterior probability of a shared causal variant (PP_H4_) ≥ 0.50 and a conditional posterior probability (PP_c_=PP_H4_/(PP_H3_+PP_H4)_) ≥ 0.80. Strong evidence for colocalization was defined as (PP_H4_) ≥ 0.80 and a conditional posterior probability (PP_H4_/(PP_H3_+PP_H4_)) ≥ 0.80.

**Figure 5:**
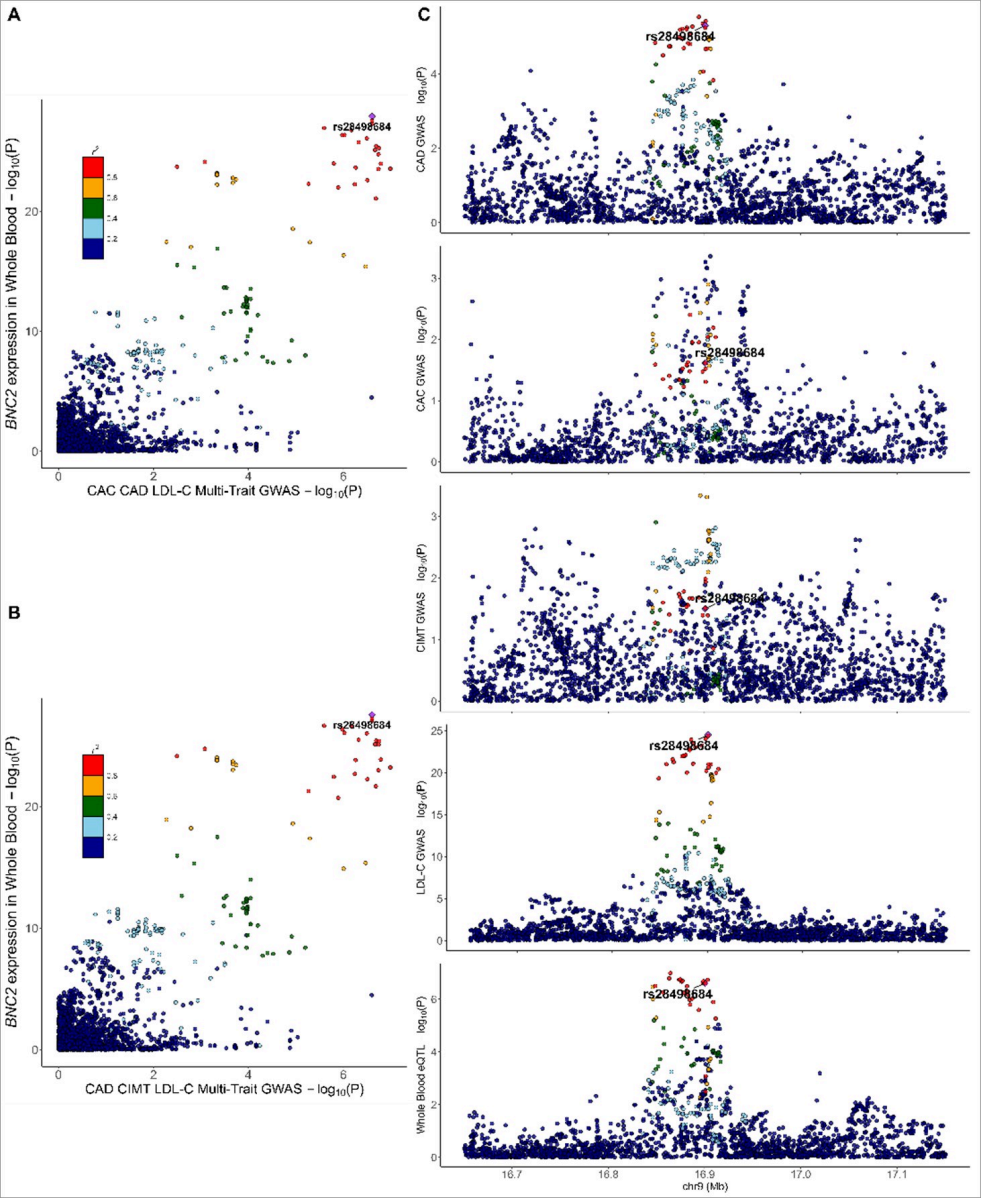
Colocalization for atherosclerosis and low density lipoprotein cholesterol with *BNC2* expression in whole blood Figure 5 illustrates the colocalization of expression for *BNC2* in whole blood with all measures of atherosclerosis and low-density lipoprotein cholesterol (LDL-C) highlighting potential shared causal variants at rs respectively. Panels A and B depict scatter plots of the gene expression in whole blood (−log10(p)) against coronary artery disease (CAD), coronary artery calcification (CAC) and LDL-C_ multi-trait GWAS results (−log10(p)), with linkage disequilibrium structure indicated by color coding. Panels C provide locus-specific association plots across CAD, CAC, CIMT GWAS and whole blood expression quantitative trait loci analyses, further supporting colocalization at these loci.

**Figure 6:**
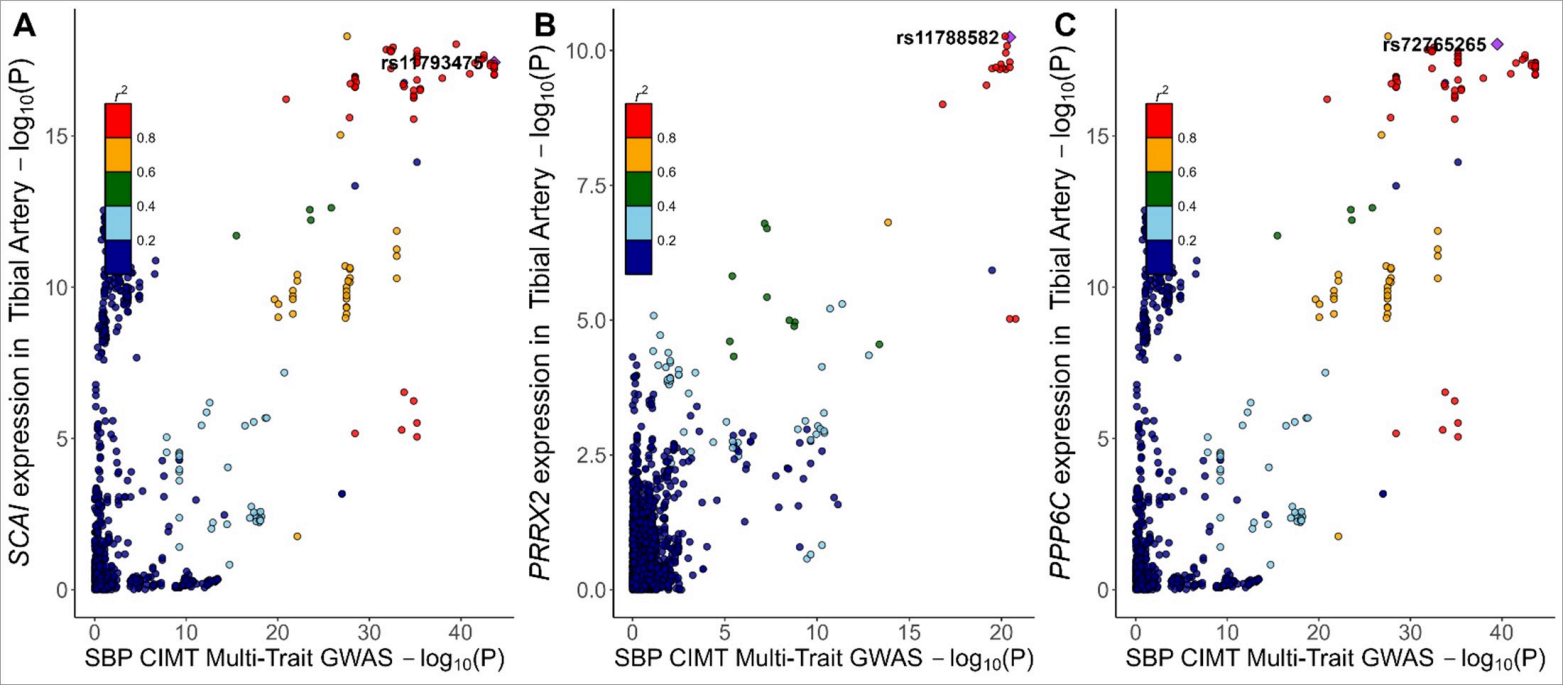
Colocalization for systolic blood pressure and carotid intima media thickness with *SCAI*, *PRRX2* and *PPP6C* expression in the Tibial Artery Figure 6 illustrates the colocalization of expression for *PRRX2*, *PPP6C* and *SCAI* in the tibial artery with systolic blood pressure (SBP) and carotid intima- media thickness (CIMT), highlighting potential shared causal variants at rs11780582 and rs11790512, respectively. Panels A B and C depict scatter plots of the gene expression in Tibial Artery (−log10(p)) against SBP-CIMT multi-trait GWAS (−log10(p)) results, with linkage disequilibrium structure indicated by color coding. Abbreviations: CIMT, C, carotid intima- media thickness; eQTL,expression quantitative trait loci, GWAS, genome-wide association study; SBP, systolic blood pressure

**Figure 7:**
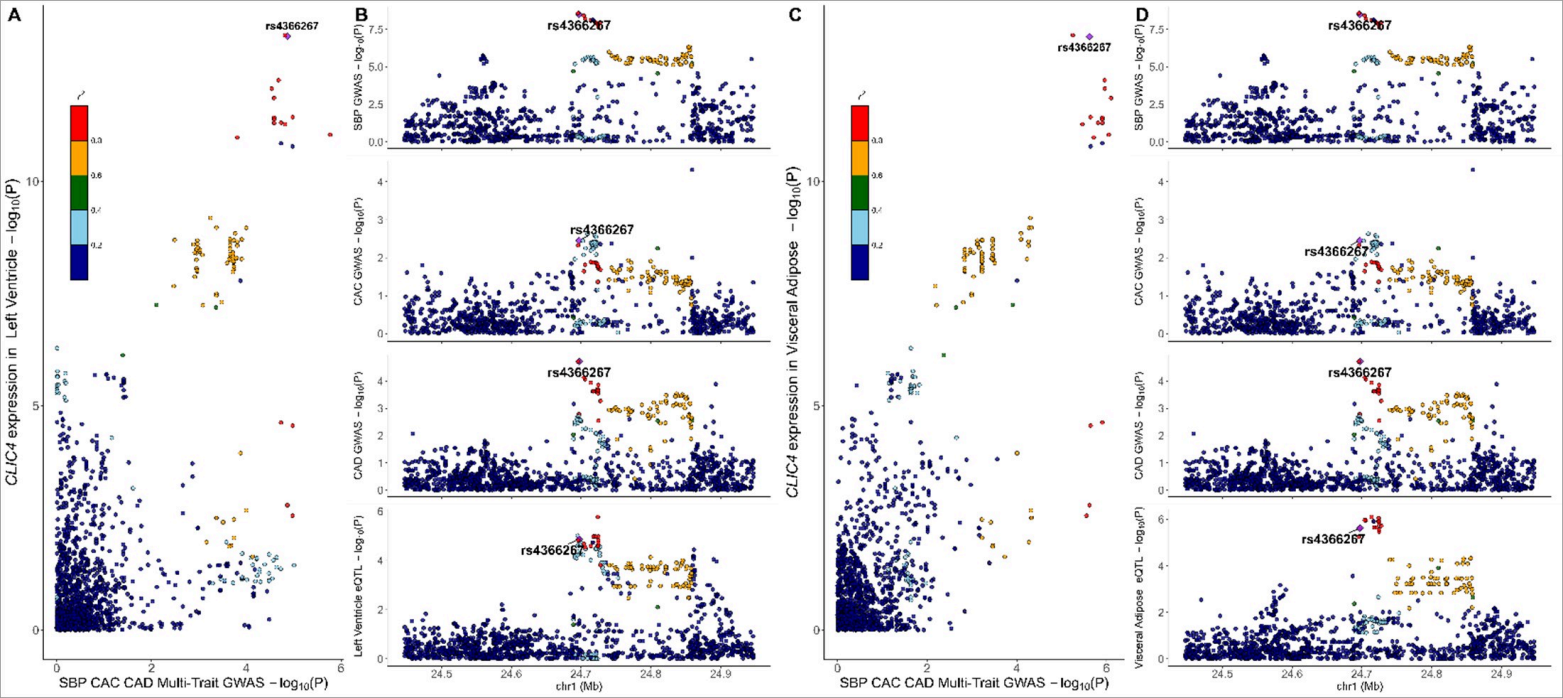
Colocalization for SBP, CAC, and CAD with *CLIC4* expression in left ventricle and visceral adipose tissue Figure 7 illustrates the colocalization of expression of *CLIC4* in left ventricle tissue and visceral adipose tissue with coronary artery disease (CAD), coronary artery calcification (CAC), and systolic blood pressure (SBP), highlighting rs as a potential shared causal variant. Panel A presents a scatter plot comparing gene expression (−log10(p)) in left ventricle heart tissue against SBP-CAC-CAD multi-trait GWAS results, with a, color-coded linkage disequilibrium structure. Panel B provides locus-specific association plots for SBP, CAC, and CAD GWAS, as well as *CLIC4* expression quantitative trait loci analyses, showing rs4366267 as a key locus across all datasets. Abbreviations: CAC, coronary artery calcification; CAD, coronary artery disease; eQTL,expression quantitative trait loci; GWAS, genome-wide association study; SBP, systolic blood pressure

We found evidence of colocalization (PP_H4_ ≥0.50 and PP_c_≥0.80) with eQTL data from GTEx with novel atherosclerosis loci. Evidence of colocalization was identified most frequently with eQTLs in adipose tissue and arterial tissue of the Aorta and Tibia (N = 132, 114, and 112, respectively). There were 18 significant novel atherosclerosis loci with evidence of colocalization with eQTL data from GTEx that had evidence of trait-trait colocalization as well (Table 1). We identified 5 genes with strong evidence for colocalization (P_R_P_A_ ≥0.80, PP_H4_ ≥0.80, and PP_c_ ≥0.80) in trait-eQTL and in trait-trait colocalization analysis (*PRRX2*, *BNC2*, *CLIC4*, *SCAI*, and *PPP6C*). SBP and CIMT colocalized with expression of *PRRX2*, *SCAI*, and *PPP6C* in arterial tissues (Fig. 5). These findings suggest a potential mechanistic link between vascular gene regulation and atherosclerosis traits. The expression of BNC2 in whole blood colocalized with CAC, CAD, CIMT, and LDL-C (Fig. 6), while the expression of *CLIC4* in visceral omentum adipose tissue and left ventricle heart tissue colocalized with CAD, CAC, CIMT, and SBP (Fig. 7).

## Discussion

Here we highlight the value of using existing, publicly available data to conduct a multi-stage, multi-trait analysis of related complex traits to understand shared genetic architecture for atherosclerosis and select risk factor traits. Furthermore, this approach allowed us to identify novel pleiotropic susceptibility loci for atherosclerosis. Specifically, we identified 195 shared loci that were novel for atherosclerosis and met our experiment-wide significance threshold, all of which underscore specific underlying pathways linked to subclinical atherosclerosis and an associated risk factor. Multi-trait colocalization further confirmed shared causal signals between atherosclerosis and selected risk factors at 25 novel atherosclerosis loci. Additionally, we integrated gene expression and eQTL data from GTEx to refine the multi-trait signals and identify functional insights for candidate genes involved in the underlying atherosclerosis pathogenesis.

Our study underscores the importance of leveraging multi-trait analysis for complex phenotypes like CAD and measures of subclinical atherosclerosis. It is well-established in the literature that CAD shares genomic risk factors with related biological traits and diseases, such as SBP and T2D.^9,12,13,24^ While single-trait GWAS continues to identify novel loci, additional resources and follow-up analyses are needed to contextualize newly discovered genetic variants. Our analysis identified 195 novel loci for atherosclerosis, 94 of which were associated with CAD, subclinical atherosclerosis, and their respective risk factors, providing further evidence for their potential roles in clinical disease. Additionally, 101 loci were identified only in analyses with CIMT or CAC and respective risk factors, suggesting a specific role in the early stages of atherosclerosis development. These findings highlight the complementary utility of subclinical traits like CIMT and CAC in uncovering potential novel genetic pathways that precede clinical disease.^19^

Importantly, we identified potential regulatory roles involved in the development of atherosclerosis by integrating tissue-specific gene expression data with novel atherosclerosis loci. We identified colocalization between the multi-trait GWAS and tissue-specific gene expression levels at 4 novel loci, involving 5 genes, including *SCAI*, *PRRX2*, and *CLIC4*. *SCAI* expression in arterial tissue colocalized with CIMT, and SBP. SCAI is a negative regulator of Rho protein activation, particularly in the RhoA/DIAPH1 pathway.^34^ Prior studies indicate that *DIAPH1* knockout in mice attenuates atherosclerosis progression, and downregulation of *DIAPH1* expression has been observed in ischemic stroke patients.^35–37^ These findings suggest that SCAI may influence both structural and functional aspects of vascular biology, warranting further investigation as a potential target for atherosclerosis research. Similarly, CIMT and SBP also colocalized with *PRRX2* expression in central and peripheral arterial tissues. PRRX2 is a transcription factor involved in vascular smooth muscle cell differentiation and migration, with established roles in cardiovascular development during embryogenesis.^38^ A recent study linked the upregulation of *PRRX2* signaling to cardiac remodeling post-myocardial infarction in a mouse model, indicating its potential involvement in SBP and CIMT through arterial vascular smooth muscle cell proliferation or remodeling.

We also identified a shared causal signal involving *CLIC4* expression in the left ventricle and visceral omentum adipose tissue, that was associated with multi-trait signals including CAD-IMT-SBP and CADCAC-SBP. *CLIC4* is implicated in apoptosis and inflammation processes that are critical to atherosclerosis development.^39–41^ Its significant colocalization with CAC, CAD, CIMT, and SBP suggests it may be a central regulator of cardiovascular and metabolic health. Emerging research using in vitro atherosclerosis cell models highlights CLIC4’s critical role in endothelial cell function and its potential as a therapeutic target for atherosclerosis.^39–42^ Future studies should explore the mechanistic pathways of *CLIC4* in immune and metabolic regulation and its therapeutic potential for atherosclerosis and hypertension.

Our study represents the first multi-trait GWAS to integrate both clinical and subclinical atherosclerosis, leveraging summary statistics from CAD, CAC, CIMT, SBP, LDL-C and T2D to enhance power for detecting novel loci associated with both early and late stages of atherosclerosis progression. This approach provides valuable insights into genetic mechanisms with potential implications for early prevention. By incorporating the largest available GWAS datasets for these traits, we offer a comprehensive perspective on their shared genetic architecture. The multi-trait framework enables the identification of pleiotropic genetic effects, refining risk loci with greater precision and uncovering shared pathways that contribute to atherosclerosis. Additionally, by integrating colocalization and functional genomic analyses, our study provides deeper biological insights linking genetic variants to gene expression and potential causal mechanisms. Addressing heterogeneity in disease progression through the inclusion of both subclinical and clinical phenotypes, our approach captures a broader spectrum of atherosclerosis development, revealing novel insights into genetic factors contributing to both early and late-stage disease, paving the way for potential early intervention and personalized prevention strategies.

Nevertheless, this our study is not without limitations. While the GWAS summary statistics include multiple population groups, non-European populations are underrepresented. Cross-population genetic correlations required the use of population-specific reference panels for genetic covariance calculations. Our sensitivity analyses demonstrated similar genetic correlations between cross-population and primarily European GWAS studies, emphasizing European ancestry representation in our dataset. Future studies would benefit from using more diverse LD reference panels and genetic correlation methods that account for multiple genetically inferred genetic ancestral groups. Additionally, the power of our multi-trait colocalization analysis was limited by differences in LD patterns, likely stemming from varying ancestry distributions in the summary statistics. Furthermore, the GWAS for subclinical traits had smaller sample sizes and larger standard errors compared to the GWAS for CAD, LDL-C, T2D, and SBP datasets. Consequently, these disparities in data quality potentially affected the multi-trait colocalization analysis, highlighting the need for larger and more ancestrally balanced datasets for future studies. Finally, we recognize that our analyses was limited to a select number of biological risk factors and may lead to an over-identification of specific biological pathways driven by SBP, T2D, and LDL-C while underrepresenting others. We selected traits based on the strength of their known relationships with not only CAD, but CAC, and CIMT, and prioritized large, well-conducted GWAS. Future studies could benefit from evaluating additional biological risk factors or disease endpoints with subclinical atherosclerosis measures to emphasize additional pathways to atherosclerosis.

In summary, our analyses identified novel loci and pathways involved in the development of atherosclerosis, underscoring the importance of subclinical traits like CIMT and CAC in uncovering early-stage mechanisms and the critical role of SBP in CAD development. Multi-trait integration revealed shared causal signals tied to vascular remodeling, inflammation, and metabolic regulation, implicating genes such as *SCAI*, *PRRX2*, and *CLIC4* as promising candidates for further research. These findings highlight the value of combining subclinical and clinical traits from publicly available GWAS data to bridge early disease processes with clinical outcomes. Future studies should replicate these discoveries in diverse populations, address ancestry-related gaps, and leverage larger datasets to enhance discovery. Nevertheless, this integrative novel approach holds promise for advancing our understanding of CAD pathogenesis and identifying novel therapeutic targets.

## Methods

We conducted a multi-stage, multi-trait analysis using publicly available GWAS summary statistics for clinical and subclinical atherosclerosis and select cardiometabolic risk factors. We leveraged the largest available CAD, CAC, and CIMT GWAS studies, which included cross-population and European ancestry GWAS, and conducted a multi-stage approach. We conducted multi-trait colocalization analysis across traits and with GTEX v8 (Fig. 1).

### Genetic Association Studies

Published GWAS summary statistics from 3 different GWAS on atherosclerosis traits (CAD, CAC, and CIMT) and 3 biological cardiometabolic risk factors (T2D, LDL-C, SBP) were collected.^11,12,14,16,19^ We selected traits for inclusion in the multi-trait analysis based on their biological relevance to atherosclerosis, the strength of their relationships with atherosclerosis, and the availability of large, high-quality GWAS datasets. To capture complementary aspects of subclinical atherosclerosis across vascular beds, we included CIMT and CAC. Information about each GWAS is available in **Supplemental Table 1**, and further details regarding the outcome measure and methods specific to each GWAS are included in the respective publications. All GWAS included well-imputed (r^2^ > 0.3) low-frequency or common variants (minor allele frequency > 1%) using either the 1000 Genomes Project, Haplotype Reference Consortium or TOPMed reference panel.^43–45^ All GWAS consisted of cross-population meta-analyses, with a majority of individuals representing individuals of European ancestry, except for the exception of the SBP GWAS. The SBP GWAS available at the time of this analysis was solely conducted in individuals with European ancestry.^12^

### Genetic correlation

Cross-trait LD score regression using LD Score v1.0 (LDSC) was used to estimate the sample overlap, genetic correlation, and shared heritability for each trait pair. LDSC evaluates genetic correlation and heritability from GWAS summary statistics using a linkage disequilibrium (LD) reference panel. We conducted the analysis using a European LD reference panel made available from the 1000 Genomes Project.^27,28,46^ As a sensitivity analysis, we repeated cross-trait LD score regression for studies that also had summary statistics available that were restricted to European populations.

#### Multi-trait analysis

We conducted a multi-stage, multi-trait analysis using N-GWAMA. N-GWAMA applies the estimates from cross-trait LD-score regression to re-weight test statistics from single-trait GWAS studies, by sample size and estimated heritability, while adjusting for genetic covariance across traits. This method enhances the detection of loci with pleiotropic associations by leveraging shared genetic architecture across traits. The newly weighted test statistics are then summated and standardized to produce a new test statistic, from which a new p-value can be calculated for each genetic variant.^27,46^ We conducted 15 multi-trait analyses using various trait combinations (Fig. 1) to identify which combination of traits led to the largest increase in associated variants with atherosclerosis and mitigate any potential power loss from the inclusion of unassociated phenotypes. Stage 1 analyses were restricted to clinical (CAD) and subclinical (CAC and CIMT) atherosclerosis traits. Stage 2 analyses included subclinical atherosclerosis traits with associated risk factors (SBP, LDL-C, T2D). Stage 3 analyses reexamined Stage 2 analyses with the inclusion of CAD (Fig. 1).

### Functional mapping and annotation

We used Functional Mapping and Annotation of GWAS (FUMA) to annotate the summary results generated from N-GWAMA.^29^ First, variants that met the GWAS significance P-value threshold (P < 5×10^−8^) were initially clumped (r^2^ < 0.6) using a non-ancestry specific LD reference panel for 1000 Genome Phase 3 to create a genomic risk locus. Within the locus, the variants were clumped again (r^2^ < 0.1) to identify independent signals. Lead variants were identified as a subset of the independent significant variants with the strongest signal, while other variants were considered secondary signals. If a lead variant was within 250 kb of another lead variant, the loci were combined. Therefore, a genomic risk locus could contain multiple lead variants. Within FUMA, ANNOVAR was used to map variants to genes by position and annotate variants according to predicted consequence and location.^47^

We reported a genomic risk locus if the lead variant from FUMA had P < 0.05 in the included single-trait GWAS summary statistics. A genomic risk locus was considered novel for atherosclerosis and the associated risk factor if the lead variant did not reach the GWAS significance P-value threshold in any of the included single-trait les. A genomic risk locus was considered novel for atherosclerosis if the lead variant did not meet the GWAS genome-wide significance threshold in the single-trait atherosclerosis summary statistics and was more than was more than ± 500 kb away from a GWAS genome-wide significant variant in the GWAS Catalog. When the analysis was restricted to subclinical atherosclerosis traits, a locus was only considered novel for atherosclerosis if it was more than ± 500 kb away from a GWAS genome-wide significant variant for CAD as well. A lead variant was considered to meet experiment-wide significance if it satisfied the Bonferroni-corrected threshold of P < 3.3×10^−9^ (5×10^−8^/15).

To identify shared and unique loci across all multi-trait analyses, we merged loci into a single shared genomic locus if the lead variants from different analyses were within 500 kb of each other. For each shared locus, the variant with the most significant P-value across analyses was designated as the shared lead variant. Shared genomic locus IDs were retained to facilitate the identification of shared regions for colocalization analyses.

### Trait-trait and Trait- eQTL colocalization

Multi-trait colocalization was conducted using HyPrColoc, a Bayesian divisive clustering algorithm designed to identify shared causal signals within a genomic region.^22^ HyPrColoc also allows the clustering algorithm to be disabled, enabling colocalization analysis across pre-defined subsets of traits, similar to COLOC and MOLOC.^31^ First, we performed trait-trait colocalization without clustering to replicate the multi-stage, multi-trait analysis conducted in N-GWAMA. Single-trait GWAS summary statistics were subset using a ± 500 kb window centered on the lead variant identified by N-GWAMA. Colocalization was then restricted to the traits included in the multi-trait GWAS analysis that identified the lead variant. Second, we conducted trait-trait colocalization across all traits using HyPrColoc’s clustering algorithm, focusing on the lead variant from the shared genomic locus. This analysis applied the variant-specific priors models with default parameters. Evidence for colocalization was determined based on the default variant-specific regional and alignment priors (P_R_*=P_A_*=0.5), with colocalization identified when P_*R*_P_*A*_≥0.25. Strong evidence of colocalization across traits was defined as P_R_P_A_≥0.80.

Finally, for loci that colocalized in the trait-trait analysis, we performed trait-eQTL colocalization using eQTL data from GTEx v8 using the COLOC package in R.^31,32 33,^ We leveraged summary statistics from the multi-trait GWAS for corresponding trait-trait colocalization and focused our analysis on a subset of GTEx v8 tissues selected based on their biological relevance to the studied traits (**Supplemental Table 8**). Colocalization was assessed using the default prior probabilities. Evidence for colocalization was defined as a posterior probability of a shared causal variant (PP_H4_) ≥ 0.50 and a conditional posterior probability (PP_c_=PP_H4_/(PP_H3_+PP_H4)_) ≥ 0.80. Strong evidence for colocalization was defined as (PP_H4_) ≥ 0.80 and a conditional posterior probability (PP_H4_/(PP_H3_+PP_H4_)) ≥ 0.80.

## Figures and Tables

**Table 1:** Novel atherosclerosis risk loci that colocalized with tissue-specific gene expression levels.

Shared Locus ID^1^	Traits in Multi-trait GWAS	Gene Symbol^2^	Tissue Type^3^	Posterior Probability (H4)^4^	Conditional Posterior Probability^5^	Candidate Causal SNP
276	CAC, CAD, LDL-C	*BNC2*	Whole Blood	0.98	0.98	rs28498684
276	CAD, CIMT, LDL-C	*BNC2*	Whole Blood	0.98	0.98	rs28498684
6	SBP, CAC, CAD	*CLIC4*	Left Ventricle	0.93	0.94	rs4366267
			Visceral Adipose	0.96	0.97	rs4366267
6	SBP, CIMT, CAD	*CLIC4*	Left Ventricle	0.92	0.94	rs72654647
			Visceral Adipose	0.97	0.97	rs6686889
287	SBP, CIMT	*PPP6C*	Tibial Artery	0.83	0.85	rs72765265
		*SCAI*	Aorta Artery	0.95	0.95	rs11793512
			Tibial Artery	0.93	0.93	rs11793475
289**	SBP, CIMT	*PRRX2*	Aorta Artery	0.98	0.98	rs11788582
			Tibial Artery	0.98	0.98	rs11788582
			Left Ventricle	0.97	0.98	rs920659
			Subcutaneous Adipose	0.80	0.84	rs920659
			Visceral Omentum Adipose	0.98	0.98	rs13299355
			Cultured fibroblasts Cells	0.98	0.98	rs59878076

Abbreviations: CAC, coronary artery calcification; CAD, coronary artery disease; CIMT. carotid intima-media thickness; GWAS, genome-wide association study; LDL-C, low- density lipoprotein cholesterol; SBP, systolic blood pressure, SNP, single nucleotide polymorphism

1 Shared Locus ID is a unique identifier for loci shared across multiple traits, defined as SNPs within 500 kb of each other.

** indicates novel for both atherosclerosis and associated risk factors.

2 HGNC Gene Symbol represents the gene whose eQTL colocalized with the genomic locus, suggesting a regulatory role.

3 Tissue indicates where the colocalized signal was detected, based on GTEx or other tissue-specific datasets.

4 Posterior Probability (H4) represents the probability that the signal is shared between traits under the H4 model of colocalization.

5 Conditional Posterior Probability reflects the posterior probability after conditioning on other signals in the locus.
